# Serum exosomes from diabetic kidney disease patients promote pyroptosis and oxidative stress through the miR-4449/HIC1 pathway

**DOI:** 10.1038/s41387-021-00175-y

**Published:** 2021-11-03

**Authors:** Chan Gao, Benyong Wang, Qi Chen, Ming Wang, Xiao Fei, Ning Zhao

**Affiliations:** Department of Nephrology, Affiliated Hangzhou First People’s Hospital, Zhejiang University School of Medicine, 310006 Hangzhou, Zhejiang China

**Keywords:** Organelles, Kidney diseases

## Abstract

**Background:**

Diabetic kidney disease (DKD) is a major contributor to end-stage renal disease. Several microRNAs (miRNAs) have been found to be enriched in exosomes of DKD patients, but it remains unclear if any of these miRNAs play an important role in the pathogenesis of DKD.

**Methods:**

Exosomes from diabetic kidney disease (DKD) patients were isolated, and the expression of miR-4449 was measured by qRT-PCR. Reactive oxygen species (ROS) was determined by DCDFA assay kit, and pyroptosis was measured by quantifying the level of activated caspase 1. mRNA and protein levels were quantified by qRT-PCR and WB.

**Results:**

In this study, we demonstrated that miR-4449 is enriched in the serum exosomes of DKD patients, and these exosomes regulate the expression of pro-inflammatory cytokines, ROS levels, and pyroptosis through miR-4449.

**Conclusions:**

Our study uncovered a novel mechanism for the progression of DKD that is mediated through miR-4449 in serum exosomes, which highlights an important role for exosomes in the pathogenesis of DKD.

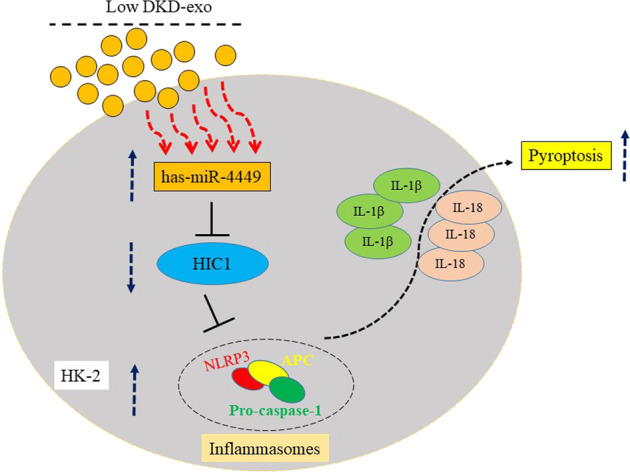

## Introduction

Diabetic kidney disease (DKD), which can lead to end-stage renal disease (ESRD), develops in 20–30% of patients with diabetes [1]. Recent studies have shown that activation of inflammatory pathways in renal tubular epithelial cells (RTECs) leads to cell death through pyroptosis, and this contributes to the pathogenesis of DKD [2]. Pyroptosis is a type of programmed cell death that involves inflammasome activation, secretion of pro-inflammatory cytokines, such as interleukin-1B (IL-1β) and interleukin-18 (IL-18), and activation of caspases [3–5]. At the molecular level, pyroptosis can be detected by the associated elevated levels of active caspase-1, GSDMD-N, a cleaved product of caspase-1, and NLRP3, an inflammasome [6]. Although the downstream effectors of pyroptosis have been well characterized, our understanding of the factors that contribute to pyroptosis in kidney cells during the pathogenesis of DKD remains incomplete.

Exosomes are small, secreted, membrane-bound organelles that contain proteins, lipids, and nucleic acids [7]. Secreted exosomes can be taken up by neighboring cells or can be transported to distant organs through body fluids, and distant cells can selectively uptake exosomes [7]. In the recipient cells, bioactive molecules that are in the cargo of exosomes can regulate signaling pathways and thus reprogram recipient cells [8]. For instance, a recent study has shown that serum exosomes present in a diabetes mouse model can transport the protein arginase 1 to aortic endothelial cells and inhibit the production of nitric oxide, thus severely affecting the function of the cells [9].

One type of biologically important cargo present in exosomes is microRNAs (miRNAs) [10]. miRNAs are short noncoding RNAs that bind to the messenger RNA (mRNA) in the 3′ untranslated region (UTR) and then alter gene expression through degradation of the mRNA or inhibition of mRNA translation [11]. miRNA is an important player in the tight regulation of gene expression under physiological conditions, and alterations in the expression of miRNAs have been shown to be involved in disease progression [12]. Interestingly, the serum of patients with diabetes has been shown to contain several miRNAs that are absent in healthy people [13, 14]. It remains unclear if these miRNAs have functional significance in the progression of DKD.

miR-4449 is one of the miRNAs that is differentially present in serum exosomes of patients with diabetic nephropathy (DN). It is overexpressed in the exosomes of DN patients compared to patients without DN [14]. However, the detailed function and molecular network of miR-4449 in DKD is still unclear. It has been acknowledged that DKD is a major reason for kidney failure, a common kidney-related complication found with type 1 diabetes mellitus and type 2 diabetes mellitus (T2DM), which is the leading cause of ESRD [15]. Previous evidence has indicated that miR-4449 is significantly correlated with the degree of albuminuria and has the potential to be used as a novel biomarker for diabetes and DN [16, 17]. Hence, investigating the biological function of DKD exosome-derived miR-4449 contributes to understanding the pathogenesis of DKD. The high concentration of glucose that leads to hyperglycemia, a condition observed in patients with diabetes, has been shown to increase the expression of hypermethylation in cancer 1 (HIC1) and its translocation to the nucleus [18]. HIC1 is a transcriptional repressor that targets genes, including SIRT1, which is a protein with antioxidant activity [18]. Thus, HIC1 plays an important role in regulating oxidative stress [18]. Oxidative stress can promote inflammation by activating transcription factors that promote the expression of pro-inflammatory cytokines [19]. In addition, oxidative stress can also activate apoptotic pathways [20]. Thus, HIC1 is an important regulator of inflammation and apoptosis, both of which are important in the pathogenesis of DKD.

Here, we investigated the functional significance of miR-4449 in regulating the expression of pro-inflammatory cytokines, reactive oxygen species (ROS) levels, and pyroptosis in RTECs. In addition, we elucidated an underlying molecular mechanism and shed light on the clinical relevance of these findings.

## Materials and methods

### Human tissues samples

Blood samples and kidney tissues of patients with DKD were from the Affiliated Hangzhou First People’s Hospital, Zhejiang University School of Medicine, Huansha Road No 261, Shangcheng District, Hangzhou, Zhejiang 310006, China. Patients with DKD were divided into three groups (*n* = 15 for each group) using the eGFR scoring method [21, 22]: high DKD (score: 89–60), middle DKD (score: 59–45) and low DKD (score: 59–45). There were 23 men and 17 women enrolled in the present study, and the ages ranged from 33 to 71 years. This research was approved by the institutional research ethical committee of the Affiliated Hangzhou First People’s Hospital, Zhejiang University School of Medicine. All experiments involving human participants were performed following the ethical standards approved by the institutional and national research committee of the Affiliated Hangzhou First People’s Hospital, Zhejiang University School of Medicine.

### Exosome isolation

Exosome precipitation solution (Exo-Quick; System Bioscience) was used to isolate exosomes. The manufacturer’s protocol was followed, with few alterations. Briefly, blood samples were drawn from the aortic sinus of patients with DKD using a sterile centrifuge tube. The samples were centrifuged at 4 °C for 10 min at 2400×*g*. The supernatants were collected, and the serum was further purified by centrifuging at 860×*g* for 10 min at 4 °C. Then, the exosomes were isolated by centrifugation at 150,000×*g* overnight at 4 °C. The pellet was dissolved in PBS, and further centrifuged at 150,000×*g* for 2 h at 4 °C. The serum exosome pellet was finally dissolved in PBS and then stored at −80 °C for subsequent use.

### Exosome uptake assay

To determine whether human kidney-2 (HK-2) can uptake DKD-exosomes, exosomes were stained with PKH67 (Sigma) following a published protocol [21]. Labeled exosomes were then co-cultured with HK-2 cells. After the indicated time of co-culture, HK-2 cells were stained with DAPI (Sigma) and visualized using confocal microscopy. Additionally, cells were examined after 24 h with confocal microscopy.

### Cell culture

Human HK-2 cells (Shanghai Biology Institute (Shanghai, P.R. China cell bank) were cultured in DMEM medium (Trueline, USA) supplemented with 10% FBS (Thermo Fisher Scientific), 2-mM l-glutamine, and 1% penicillin/streptomycin (Solarbio, P.R. China). The cells were incubated at 37 °C in a 5% CO_2_ atmosphere. N-acetyl cysteine (NAC; S1623, Selleck, USA) was dissolved in DMSO (D2650, Sigma, USA) and was added in the culture media to a final concentration of 10 μM where indicated.

### Quantitative reverse transcription polymerase chain reaction (qRT-PCR)

TRIzol Reagent (Invitrogen, USA) was used to extract total RNA, and a cDNA synthesis kit (Thermo Fisher Scientific, USA) was used to synthesize cDNA. RNA levels were determined using qRT-PCR and were normalized to U6 for microRNA and to GAPDH for mRNA. An average of three replicates was used for each data point. The following are the primers used in the study: hsa-miR-4449, F: 5′-CGTCCCGGGGCTGCGC-3′, R 5′-AGTGCAGG GTCCG AGGTATT-3′; U6, F: 5′-CTCGCTTCGGCAGCACA-5′, R 5′-AACGCTTCACG AATTTGCGT-5′; HIC1, F: 5′-TCGTGCGACAAGAGCTAC-3′, R: 5′-ACTTCTTCCCGC AGATGG-3′; GAPDH, F: 5′-AATCCCATCACCATCTTC-3′, R 5′-AGGCTGTTG TCATA CTTC-3′. Three replicates were necessary for each reaction.

### Western blotting (WB)

Whole cell extracts were prepared by lysing the cells in RIPA buffer (JRDUN, P.R. China) supplemented with EDTA-free protease inhibitor Cocktail (Roche, Germany). For each sample, 25 μg of protein were fractionated on an SDS-PAGE gel. The proteins were then transferred to a nitrocellulose membrane (Millipore, USA). The membrane was blocked with 5% nonfat dry milk for 1 h at room temperature and probed with primary antibodies overnight at 4 °C followed by 1 h incubation at 37 °C with secondary antibodies (1:1000; Beyotime, P.R. China). An enhanced chemiluminescence system (Tanon, P.R. China) was used for visualization. The primary antibodies were: CD63 (Ab134045, Abcam, UK), CD81 (ab109201, Abcam, UK) and TSG101 (ab125011, Abcam, UK), HIC1 (ab49326, Abcam, UK); NLRP3 (ab263899, Abcam, UK); Active Caspase-1 (#4199, CST, USA); Pro-Caspase-1 (#2225, CST, USA); GSDMD-N (ab215203, Abcam, UK); and GAPDH (#5174, CST, USA). Three replicates were necessary for each reaction.

### Knockdown and overexpression

Three siRNAs targeting different portions of human HIC1 (NM_001098202.1) and a scramble siRNA (siNC: 5′-CAGUACUUUUGUGUAGUACAA-3′) were cloned in pLKO.1 lentiviral plasmids. The human HIC1 coding sequence was cloned into pLVX-puro vectors. Plasmids were transfected into cells using Lipofectamine 2000 (Thermo Fisher Scientific) for transient overexpression. All the experiments were performed 48 h after transfection. The HIC1 siRNA sequences were: siHIC1-1: (1391–1409; 5′-GCUUCGGUGACAACCUGUATT-3′), siHIC1-2: (1712–1730; 5′-CCAUCUGC GGGAAGAAGUUTT-3′), siHIC1-3 (1914–1932, 5′-CCUCAUCAGCCACAUGAA GTT-3′).

### Enzyme-linked immunosorbent assay (ELISA)

IL-1β and IL-18 expressions were measured using commercial IL-1β and IL-18 quantitative ELISA kits following the manufacturer’s protocol. Briefly, the IL-1β and IL-18 antibodies were incubated in an ELISA plate for 2 h at 37 °C. The plate was washed with scrubbing solution five times before adding the secondary antibodies. Finally, the stop solution was added to the plate, and OD450 values were measured in each well within 5 min using a microplate reader (Pulangxin, China). For each data point, three experiments were performed.

### Cell pyroptosis assay

Pyroptosis was assessed by quantifying caspase-1 activation using a FAM-FLICA Caspase-1 Assay Kit (ImmunoChemistry Technologies, USA). Cells were incubated with a caspase-1 detection probe for 1 h in the dark after stimulating with SEA for 24 h. The cells were washed with the washing buffer and then stained with propidium iodide (PI) for 20 min. Afterward, the cells were analyzed using flow cytometry (BD FACSCalibur, USA). Cleaved caspase-1 and PI double positive cells were defined as pyroptotic cells. Three replicates were necessary for each reaction.

### ROS measurement

A DCFDA assay kit was used to measure cellular ROS. Briefly, the cells were excited with a 488 nm wavelength, and emission at a 520 nm wavelength was measured using a Wallac 1420 microplate reader (PerkinElmer, USA). The DCF fluorescence intensity indicated the relative amount of intracellular ROS. Three replicates were necessary for each reaction.

### Dual luciferase assay

TargetScan and Starbase were used to predict the binding sites of miR-4449 and HIC1. The wild type and mutant sequences in the 3′UTR of HIC1 were cloned into pGL3-basic luciferase reporter vectors. The plasmids were co-transfected with miR-4449 inhibitor or mimic into HK-2 cells, and the luciferase expression was quantified using a dual luciferase reporter gene kit (Beijing Yuanpinghao Biotechnology Co., Ltd.). Three replicates were necessary for each reaction.

### Statistical analysis

GraphPad Prism software Version 7.0 (La Jolla CA, USA) was used for all statistical analyses. At least three independent experiments were performed for each data point, and the data were presented as the mean ± SD. Student’s *t*-test was used to calculate statistical significance when two groups were compared, and a one-way analysis of variance was used to calculate the statistical significance when multiple groups were compared. Statistical significance was defined as *P*-value < 0.05.

## Results

### miR-4449 is upregulated in serum exosomes derived from patients with DKD

**Fig. 1:**
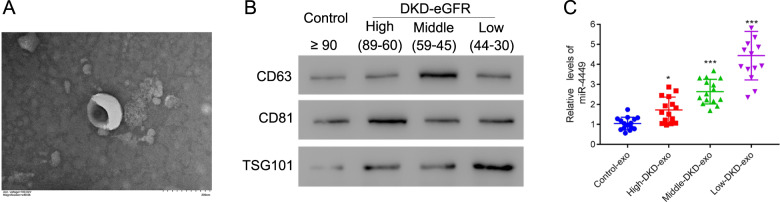
miR-4449 is upregulated in serum exosomes of patients with DKD. Patients with DKD were divided into three groups (*n* = 15 for each group) using the eGFR scoring method: high DKD (score: 89–60), middle DKD (score: 59–45), and low DKD (score: 59–45). Exosomes were isolated from the serum of the patients. **A** The structure of the exosomes was imaged with transmission electron microscopy (TEM). **B** Protein levels of exosome markers from patients with different degrees of DKD were examined with Western blotting using the indicated antibodies. **C** The miR-4449 levels in different DKD exosomes were determined using RT-PCR. *P < 0.05 vs. Control-exo, ***P < 0.001 vs. Control-exo.

To test the potential role of miRNAs enriched in serum exosomes derived from patients with DKD, we measured the level of miR-4449 in the serum of patients with DKD. Exosomes were isolated, and the integrity of the exosomes was verified through transmission electron microscopy (Fig. 1A). As expected, the isolated exosomes contained proteins that are known to be enriched in exosomes, such as CD63, CD81, and TSG101 (Fig. 1B). Next, we examined the expression of miR-4449 using qRT-PCR. Indeed, patients with DKD had significantly higher levels of miR-4449 in serum exosomes compared with those of controls (Fig. 1C). In addition, miR-4449 levels increased with disease severity (Fig. 1C).

### HK-2 cells uptake exosomes from DKD patients

**Fig. 2:**
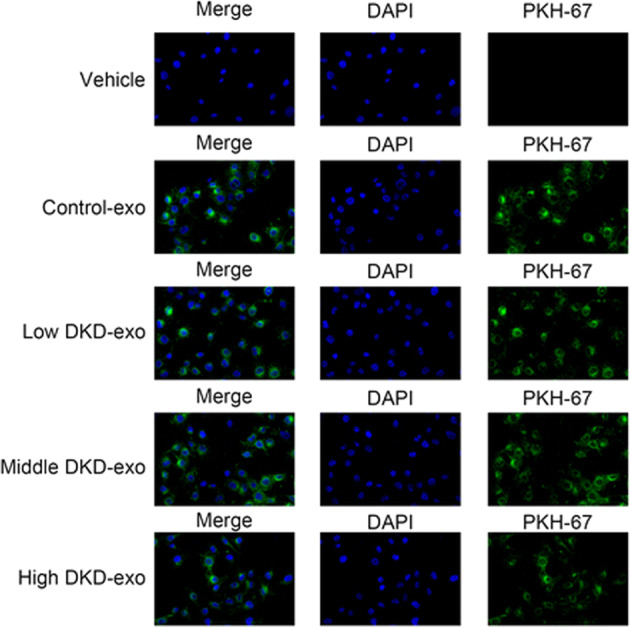
Human kidney cells uptake exosomes from patients with DKD. **A** Exosomes from patients with DKD were incubated with PKH-67 and HK-2 cells were treated with these exosomes. Uptake of exosomes by HK-2 cells was visualized with confocal microscopy.

The kidney is the primary organ affected by diabetes, so we tested whether kidney cells were able to uptake serum exosomes obtained from patients with DKD. We labeled serum exosomes with PKH-67 dye (50 µg) and examined the intake of these exosomes by HK-2 cells, which is a proximal tubular renal cell line. Indeed, the HK-2 cells showed the incorporation of PKH-67 dye inside the cells (Fig. 2A), indicating that proximal renal epithelial cells uptake serum exosomes.

### DKD exosomes promote ROS accumulation and pyroptosis in kidney cells

**Fig. 3:**
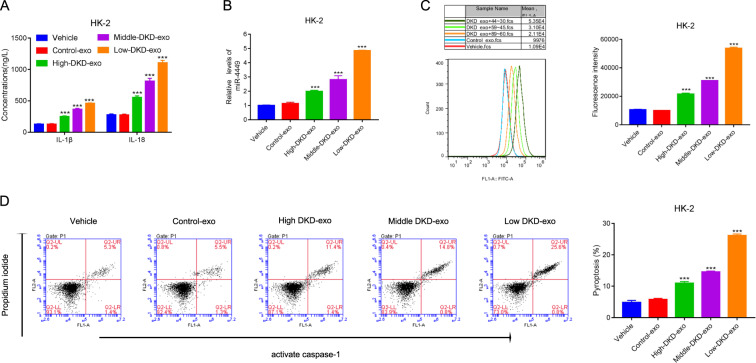
DKD exosomes promote ROS accumulation and pyroptosis in kidney cells. HK-2 cells were treated with exosomes isolated from patients with DKD with different degrees of disease progression and controls. **A** The secretions of IL-1β and IL-18 were measured using ELISA. **B** The relative level of miR-4449 was measured with RT-PCR. **C** The reactive oxygen species (ROS) level was measured using a DCFDA assay kit. **D** Pyroptosis was measured through quantification of cells with active caspase-1. ***P < 0.001 vs. control-exo.

Next, we examined the effect of serum exosomes isolated from patients with DKD on HK-2 cells. First, we checked the immune response in the cells upon exposure to DKD exosomes (50 µg). The expressions of IL-1β and IL-18 were significantly higher in cells incubated with DKD exosomes compared with those in control cells (Fig. 3A). In addition, this increase was positively correlated with the severity of DKD (Fig. 3A). As expected, the expression of miR-4449 in HK-2 cells was significantly higher in cells incubated with DKD exosomes, and the expression levels were positively correlated with the severity of DKD (Fig. 3B).

We also investigated the level of ROS in HK-2 cells in the presence of exosomes from patients with DKD and controls and found that cells treated with DKD exosomes had significantly higher ROS levels compared with cells treated with control exosomes or vehicle (Fig. 3C). Furthermore, this increase in ROS levels was positively correlated with the severity of DKD (Fig. 3C).

Finally, we examined the effect of the increase in the expression of pro-inflammatory genes and ROS levels on pyroptosis. To measure pyroptosis, we quantified the levels of activated caspase-1 in cells treated with DKD serum. Indeed, cells cultured with DKD exosomes showed significantly higher levels of active caspase-1 (Fig. 3D), indicating a higher level of pyroptosis in the cells exposed to DKD exosomes.

**Fig. 4:**
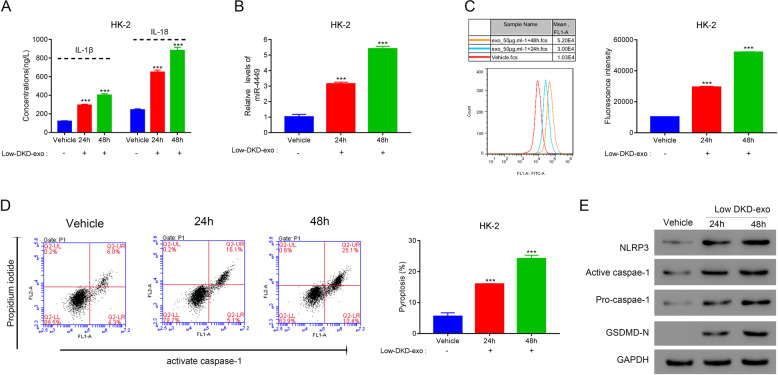
DKD exosomes promote ROS accumulation and pyroptosis in kidney cells in a time-dependent manner. Exosomes from patients with low-DKD were isolated, and HK-2 cells were treated with these exosomes for 24 and 48 h. **A** The secretions of IL-1β and IL-18 were quantified using ELISA. **B** The relative level of miR-4449 was quantified with RT-PCR. **C** ROS levels were quantified using the DCFDA assay kit. **D** Pyroptosis was measured through quantification of cells with active caspase-1. **E** Expression of indicated proteins was measured by western blotting. ***P < 0.001 vs. vehicle.

In addition, IL-1β and IL-18 expressions were significantly higher in cells treated with DKD exosomes for 48 h compared with a 24 h-treatment (Fig. 4A). Likewise, the cells treated with DKD exosomes for 48 h also had significantly higher levels of miR-4449 (Fig. 4B), significant increases in ROS levels (Fig. 4C), and significantly more pyroptosis compared with cells treated with DKD exosomes for 24 h (Fig. 4D), indicating a time-dependent response to DKD exosome treatment. We also assessed the expression of active caspase-1, GSDMD-N, and NLRP3, and found that their expressions were higher after 48 h of DKD exosome treatment compared with a 24 h treatment (Fig. 4E).

**Fig. 5:**
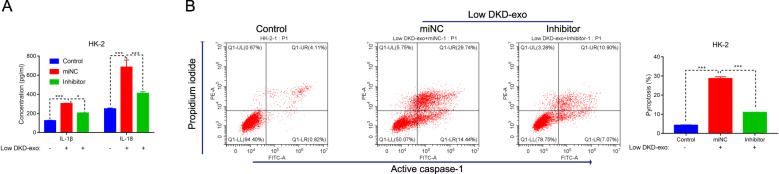
Has-miR-4449 inhibitor disrupted the function of Low DKD-exo in the pyroptosis of HK-2 cells. **A** The has-miR-4449 inhibited suppression of the secretions of IL-1β and IL-18 in HK-2 cells cultured with Low DKD-exo, ***P < 0.001. **B** The pyroptosis of HK-2 cells that were cultured with low DKD-exo were deeply suppressed in the presence of the miR-4449 inhibitor. ***P < 0.001.

To further determine the connection between DKD-exosome and miR-4449, the miR-4449 inhibitor and low DKD-exo were used to co-culture human HK-2 cells. As shown in Fig. 5A, the secretions of IL-1β and IL-18 were deeply reduced in low DKD-exo cultured cells in the presence of the miR-4449 inhibitor. Moreover, the miR-4449 inhibitor also suppressed the pyroptosis of low DKD-exo cultured cells (Fig. 5B). Together, these results suggested that low DKD-exo promoted the pyroptosis of HK-2 by delivering miR-4449. Inhibiting the activity of miR-4449 deeply abolished the function of low DKD-exo in pyroptosis of human HK-2 cells.

### HIC1 suppresses ROS levels and pyroptosis induced by DKD exosomes in kidney cells

**Fig. 6:**
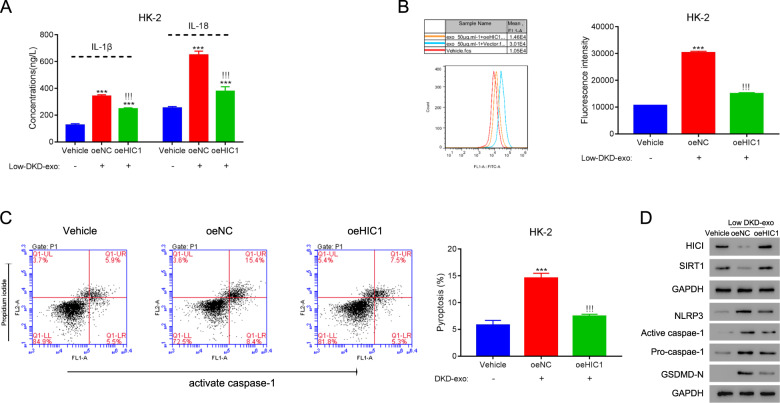
HIC1 suppresses ROS levels and pyroptosis caused by DKD exosomes in kidney cells. HK-2 cells transfected with vehicle, empty vectors (oeNC), or HIC1 plasmids (oeHIC1) were treated with exosomes isolated from low-DKD patients for 24 h. **A** The secretions of IL-1β and IL-18 were quantified using ELISA. **B** ROS levels were quantified using the DCFDA assay kit. **C** Pyroptosis was measured by quantifying cells with active caspase-1. **D** Expression of indicated proteins was measured by western blotting. ***P < 0.001 vs. vehicle, !!!*P* < 0.001 vs. oeNC.

We investigated the mechanism through which DKD exosomes regulate ROS levels and pyroptosis in RTECs. HIC1 has been shown to mediate ROS accumulation under high glucose conditions in RTECs [18]. Hence, we tested whether DKD exosome-mediated effects on ROS accumulation and pyroptosis occur through HIC1. Overexpression of HIC1 significantly reduced the effects of DKD exosomes on IL-1β and IL-18 expressions (Fig. 6A). Likewise, HIC1 overexpression attenuated the effect of DKD exosomes on ROS levels (Fig. 6B). As expected, HIC1 overexpression also significantly decreased the level of active caspase-1 in DKD exosome treated cells, and pyroptosis was significantly reduced upon HIC1 overexpression (Fig. 6C). In addition, HIC1 overexpression lowered the protein levels of active caspase-1, NLRP3, and GSMD-N (Fig. 6D).

### miR-4449 inhibits expression of HIC1

**Fig. 7:**
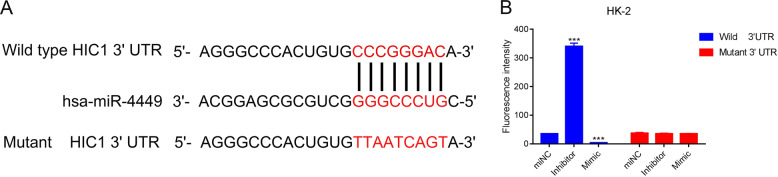
miR-4449 inhibits expression of HIC1. **A** Sequences of wild type HIC1 3′UTR (wild type), miR-4449 and HIC1 3′UTR with mutations in the sequence complementary to miR-4449 (mutant) are shown. **B** HK-2 cells transfected with reporter dual luciferase gene with wild type or mutant 3′UTR of HIC1 were treated with control miRNA (miNC), miR-4449 inhibitor (inhibitor), or miR-4449 mimic (mimic), and expression of luciferase was quantified. ***P < 0.001 vs. miNC.

To identify the downstream mediator of miR-4449 in regulating pyroptosis, we used the TargetScan database, which can be used to predict potential targets of miRNAs [23]. The 3′UTR of HIC1 was identified as a potential binding site of miR-4449 (Fig. 7A), suggesting that HIC1 could be a target of miR-4449. To test if miR-4449 affects the expression of HIC1 by targeting the complementary sequence in the 3′UTR of HIC1, we developed the following reporter system: the 3′UTR of HIC1 (wild type, WT HIC1 3′UTR) or the 3′UTR of HIC1 with a mutation in the complementary sequence of miR-4449 (mutant HIC1 3′UTR; Fig. 7A) were cloned downstream of a luciferase reporter, and the expression of the luciferase gene was examined in the presence of a miR-4449 inhibitor and miR-4449 mimic. Inhibition of miR-4449 caused a significant increase in luciferase expression, whereas expression of the miR-4449 mimic led to a significant decrease in the expression of luciferase in cells transfected with WT HIC1 3′UTR (Fig. 7B). On the other hand, neither the miR-4449 inhibitor nor the miR-4449 mimic affected the expression of luciferase in mutant HIC1 3′UTR transfected cells (Fig. 7B).

### HIC1 overexpression rescues the effect of miR-4449

**Fig. 8:**
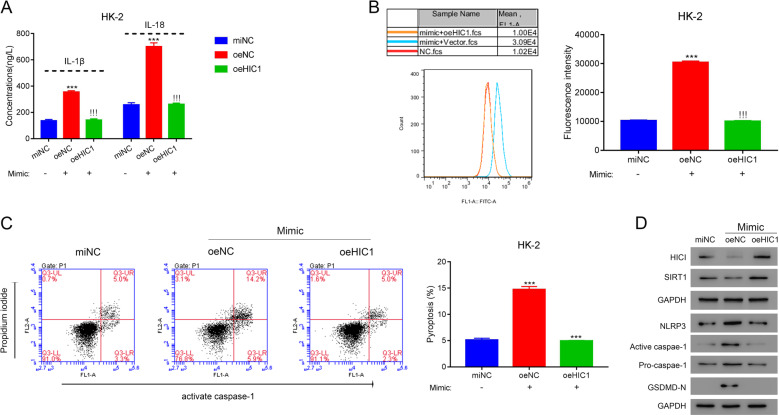
HIC1 overexpression rescues the effect of miR-4449. HK-2 cells transfected with vehicle, empty vectors (oeNC), or HIC1 plasmid (oeHIC1) were treated with control miRNA (−) or miR-4449 mimic (mimic). **A** The secretions of IL-1β and IL-18 were quantified using ELISA. **B** ROS levels were quantified using the DCFDA assay kit. **C** Pyroptosis was measured by quantifying cells with active caspase-1. **D** Expression of indicated proteins was measured by western blotting. ***P < 0.001 vs. miNC; !!*P* < 0.001 vs. oeNC.

To further elucidate the functional interaction between miR-4449 and HIC1, we examined the effect of HIC1 overexpression in miR-4449 mimic-expressing cells. Indeed, overexpression of HIC1 in cells that were transfected with an miR-4449 mimic blunted the effect of the miRNA mimic on IL-1β and IL-18 expressions (Supplementary Fig. 1A, B and Fig. 8A). Likewise, miR-4449 mimic expression did not cause a significant difference in the ROS levels (Fig. 8B) and pyroptosis (Fig. 8C) in cells overexpressing HIC1. Furthermore, levels of active caspase-1, NLRP3, and GSDMD-N were reduced upon HIC1 overexpression in cells expressing the miR-4449 mimic (Fig. 8D).

### HIC1 inhibits pyroptosis by reducing oxidative stress

**Fig. 9:**
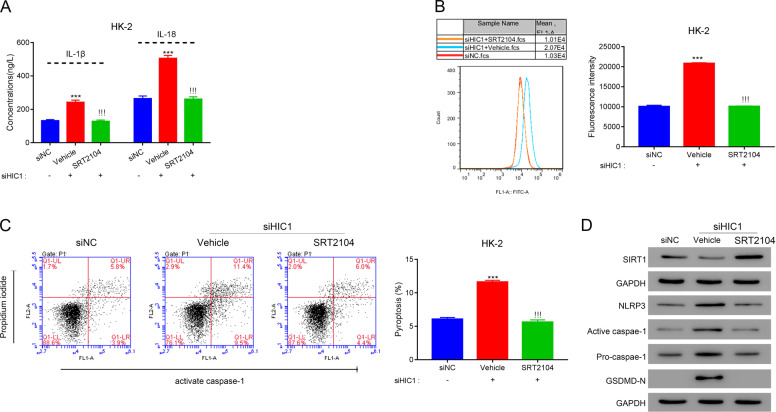
HIC1 inhibits pyroptosis by reducing oxidative stress. HK-2 cells were transfected with control siRNA (siNC) or HIC1 siRNA (siHIC1), and the cells were treated with a vehicle or N-acetyl cysteine (NAC). **A** The secretions of IL-1β and IL-18 were quantified using ELISA. **B** ROS levels were quantified using the DCFDA assay kit. **C** Pyroptosis was measured through quantification of cells with active caspase-1. **D** Expression of indicated proteins was measured with western blotting. ***P < 0.001 vs. siNC; !!!*P* < 0.001 vs. vehicle.

Next, we investigated the mechanism through which HIC1 regulates inflammation, ROS levels, and pyroptosis. HIC1 has been shown to regulate ROS levels by transcriptionally silencing SIRT1 [18]; hence, we tested whether HIC1 regulates pyroptosis by lowering oxidative stress. We examined the effect of treatment with NAC, a potent antioxidant, on HK-2 cells with HIC1 knockdown. Knockdown of HIC1 significantly increased IL-1β and IL-18 expressions (Supplementary Fig. 1C, D and Fig. 9A); however, knockdown did not affect the expression of these cytokines in the cells that were treated with NAC (Fig. 9A). Likewise, the ROS levels were significantly increased in HIC1 knockdown cells treated with a vehicle, but not in the cells treated with NAC (Fig. 9B). A similar effect of NAC on pyroptosis was also observed (Fig. 9C). Furthermore, NAC reversed the effect of HIC1 knockdown on the level of active caspase-1, NLRP3, and GSDMD-N (Fig. 9D).

### miR-4449 expression is negatively correlated with HIC1 in DKD patients

**Fig. 10:**
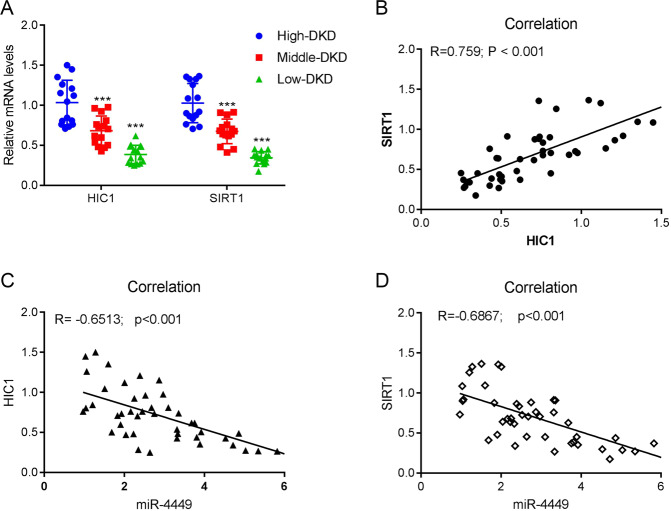
miR-4449 expression was negatively correlated with the transcriptomic levels of HIC1 in patients with DKD. RNA from kidney tissues of patients with DKD was isolated. **A** The relative mRNA level of HIC1 in patients with DKD at different stages was measured with qRT-PCR. **B** The correlation between the expression of HIC1 and miR-4449 was plotted. ***P < 0.001 vs. high-DKD.

Finally, we explored whether the miR-4449-mediated molecular mechanism observed in HK-2 cells had clinical relevance. We isolated RNA from the kidney tissue of patients with DKD and measured the expressions of miR-4449 and HIC1. The expression of HIC1 was significantly reduced as the disease progressed (Fig. 10A). In addition, HIC1 expression was negatively correlated with the expression of miR-4449 in patients with DKD (Fig. 10B), which was consistent with the observed effect of miR-4449 on the expression of HIC1 in HK-2 cells.

## Discussion

In this study, we demonstrated that serum exosomes from patients with DKD increased the expression of pro-inflammatory cytokines, the level of ROS, and pyroptosis in RTECs. This is the first evidence that linked an miRNA present in serum exosomes directly to the pathogenesis of DKD. This finding highlights the critical role that exosomes play in the pathogenesis of DKD, and thus establishes exosomes as a new contributor to DKD.

We established that miR-4449 is a cargo of serum exosomes in patients with DKD, and its levels increase as the disease progresses. We also demonstrated that miR-4449 regulates IL-1β and IL-18 expressions, the level of ROS, and pyroptosis, and thus plays a critical role in DKD pathogenesis. A recent study has identified several miRNAs enriched in the serum of patients with T2DM with DKD or without complications [24]. In this study, 38 miRNAs were found to be differentially present in the patients with T2DM compared with their healthy counterparts; however, miR-4449 was not one of the miRNAs identified in this study [24]. It is possible that miR-4449 is enriched specifically in exosomes and might not have been detected in the study. miR-99b and miR-122-5p were found to be enriched in patients with DKD compared with T2DM patients without complications and healthy patients [24]. However, the biological significance of this enrichment in the pathogenesis of DKD was not explored in the study. Our study identified miR-4449 as an miRNA that not only correlates with, but is also involved with, the pathogenesis of DKD.

We identified HIC1 as a functional target of miR-4449 and demonstrated that HIC1 expression is suppressed by miR-4449. Previous studies have shown that HIC1 is activated by high glucose, and it contributes to oxidative stress by repressing the expression of SIRT1, an antioxidant gene. This study identified miR-4449 as an upstream regulator of HIC1, and thus highlights the important role of miR-4449 in the pathogenesis of DKD.

The kidney is the primary organ affected by diabetes, and DKD remains a clinically challenging problem that accounts for the majority of ESRD. Hence, effective therapeutic targets for DKD are still needed. Cell death in the kidney is a major contributor to the progression of DKD [25]. In this study, we showed that serum exosomes as well as miR-4449 increase the level of ROS and pyroptosis in RTECs, both of which can cause cell death. Furthermore, we showed that serum exosomes and miR-4449 promote pro-inflammatory cytokine expression, and inflammation is known to be a factor that contributes to DKD [26]. Hence, our findings suggest that serum exosomes and miR-4449 as well as the downstream mediator of miR-4449; i.e., HIC1, are potential targets for the treatment of DKD. We further showed that NAC, a commonly used dietary supplement, can counteract the effect of miR-4449 on the expression of pro-inflammatory genes, ROS levels, and pyroptosis. Thus, our study suggests a beneficial role of dietary antioxidants in counteracting the progression of DKD. Future epidemiological research on patients with DKD could validate the clinical benefits of dietary antioxidants in patients with DKD.

Our study demonstrated that miRNAs present in serum exosomes of patients with DKD contribute to DKD pathogenesis. It is possible that miRNAs present in the serum of patients with other non-diabetic diseases could be an important in the pathogenesis of these diseases. Although the number of clinical samples limited the significance of our finding, our results lay a foundation for future studies to investigate the clinical significance of serum exosome miRNAs in the context of other diseases. Therefore, a large study with more clinical samples is required to confirm our findings in subsequent research.

## Supplementary information

Supplementary figure files

## Data Availability

The datasets used during the current study are available from the corresponding author on reasonable request.
