# Supplementary material for: Serum exosomes from diabetic kidney disease patients promote pyroptosis and oxidative stress through the miR-4449/HIC1 pathway
Source: Nutr Diabetes. 2021 Nov 3;11:33. doi: 10.1038/s41387-021-00175-y (PMC8566490; doi:10.1038/s41387-021-00175-y)
Supplement: Supplementary file 1 — Supplementary figure files [file 41387_2021_175_MOESM1_ESM.docx]

**
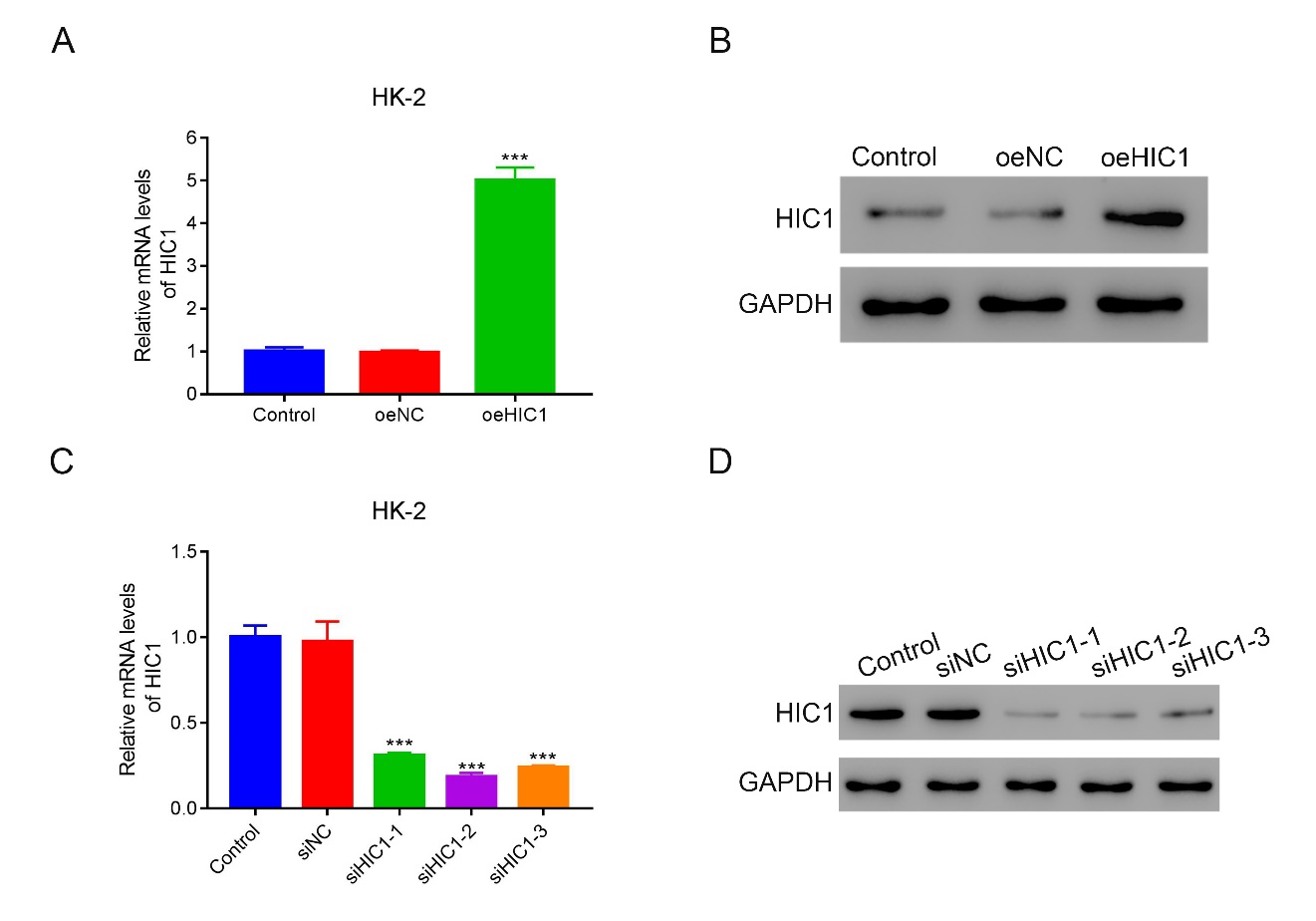
**

**Supplementary Fig. 1: Overexpression and silencing of HIC1 in HK-2 cells. A and B.** HK-2 cells were transfected with an empty vector (oeNC) or HIC1 encoding plasmid (oeHIC1). (A) the relative mRNA level of HIC1 was measured with RT-PCR, and (B) protein levels were measured with western blotting using the indicated antibodies. *** indicates p < 0.001 vs. oeNC. **C and D.** HK-2 cells were transfected with control siRNA (siNC) or HIC1 siRNA (siHIC1-1,2,3), (C) the relative mRNA of HIC1 was measured with RT-PCR, and (D) the protein levels of HIC1 were measured with western blotting using the indicated antibodies. *** indicates p < 0.001 vs. siNC.
